# Isokinetic Trunk Strength in Acute Low Back Pain Patients Compared to Healthy Subjects: A Systematic Review

**DOI:** 10.3390/ijerph18052576

**Published:** 2021-03-04

**Authors:** Waleska Reyes-Ferrada, Luis Chirosa-Rios, Angela Rodriguez-Perea, Daniel Jerez-Mayorga, Ignacio Chirosa-Rios

**Affiliations:** 1 Department Physical Education and Sports, Faculty of Sport Sciences, University of Granada, 18011 Granada, Spain; waleska.reyes@unab.cl (W.R.-F.); lchirosa@ugr.es (L.C.-R.); angrp91@gmail.com (A.R.-P.); ichirosa@ugr.es (I.C.-R.); 2 Faculty of Rehabilitation Sciences, Universidad Andres Bello, Viña del Mar 2531015, Chile; 3 Faculty of Rehabilitation Sciences, Universidad Andres Bello, Santiago 7591538, Chile

**Keywords:** dynamometer, core muscles, trunk strength testing, reference data, peak torque

## Abstract

Background: The purpose of this systematic review was to: (I) determine the quality of evidence from studies assessing trunk isokinetic strength in subjects with acute low back pain (ALBP) compared to healthy subjects and (II) establish reference values of isokinetic trunk strength in subjects with ALBP. Methodology: Preferred Reporting Items for Systematic Review and Meta-Analyses (PRISMA) statements were followed using keywords associated with trunk, strength and low back pain. Four databases were used: PubMed, Web of Science, Scopus and SPORTDiscus. Methodological quality was assessed using the Quality Assessment of Diagnostic Accuracy Studies (QUADAS). Results: A total of 1604 articles were retrieved, four included in this review. All were evaluated as high risk of bias (Rob). Due to the high Rob and the diversity of protocols, instruments and variables used, it was not possible to determine reference values for subjects with ALBP, we can only establish a range of flexion peak torque (PT) between 175.1 and 89.7 Nm at 60°/s and between 185 and 81.5 Nm at 120°/s, and for extension PT between 240.0 and 91.5 Nm at 60°/s and between 217.5 and 69.2 Nm at 120°/s in subjects with ALBP. Conclusions: Due to the low quality of the evidence and the diversity of protocols used when measuring trunk isokinetic strength, it is necessary to carry out new high-quality research to establish reference values of trunk strength in subjects with ALBP.

## 1. Introduction

Low back pain (LBP) is among the three leading causes of years lived with disability [1], only in 2017 577 million people suffered from LBP [2]. LBP refers to pain, muscle tension or stiffness below the costal border and over the lower gluteal fold, with or without sciatia. It can be classified according to its duration in acute low back pain (ALBP), less than six weeks, or chronic low back pain (CLBP) when the pain persists for more than three months [3]. It is estimated that 80% of the population will suffer from LBP at least once in their lives [4,5], but these symptoms should disappear within six weeks. Although a significant number of patients will have recurrences or persistent pain and disability [6,7], even in the follow-up to one year, some patients will still show mild to moderate levels of pain and disability [8]. Da Silva et al. [9] reports a pain episode recurrence in 70% of the patients within 12 months after recovery from the first ALBP episode, of which 40% will suffer a moderate functional limitation or will need to use the health system, suggesting that the good prognosis of ALBP has been overestimated. 

Regarding the cause of the LBP, it is not often possible to determine an anatomical source of pain (e.g., epidural abscess, compression fracture, spondyloarthropathy, malignancy or cauda equina syndrome) [10]. Most of the times, in 90% of cases, no specific cause is identified for which it is denominated non-specific LBP (NSLBP) [10]. However, multiple factors have been associated with the occurrence of NSLBP, among them the alteration of the neuromuscular response of the trunk [11,12], the deconditioning (or decrease in the function) of the lumbar musculature [13,14], the reduction in the muscular mass of the trunk [15], and the reduction in the muscular strength of the trunk [13,16,17].

The spine needs to be mechanically stable at all times to avoid injuries that can eventually lead to pain [18]. Maintaining this stability is role of the active neuromuscular system [19], and thus the trunk strength plays an important role in different aspects related to health and sport [20,21,22,23]. The trunk is the center of the kinematic chains, transferring forces and acting as a bridge between the upper and lower extremities [24]. Arms and legs can be compared with their contralateral to define deficits or imbalances but, unlike the extremities, the trunk does not have this possibility, which makes it difficult to find parameters of normality or reference. Trunk strength has been related to injury prevention [25,26], which is why it plays an important role in the functional evaluation of people or athletes [27,28].

To evaluate trunk strength, several methods have been developed. The gold standard is the isokinetic dynamometry, which consists of measuring muscle strength capacity under linear or rotational movements at constant velocities [29]. This method allows a quick quantification of several muscle function parameters at different positions and angular velocities, and its use has been recommended for clinical and research purposes [30].

Prospective studies have shown that trunk strength imbalance [17] and decreased trunk muscle strength could be considered risk factors for developing NSLBP, specifically isometric and isokinetic strength of trunk flexors and lumbar extensors muscles [16]. To the best of our knowledge, there are no reference values in the development of the first episode of ALBP; instead, the evidence shows that, when comparing healthy subjects with CLBP patients, the lumbar extensor peak torque is lower, but the flexor peak torque does not decrease in the same way, so the ratio flexors/extensors (F/E) do not decrease [31]. These data are important since the parameters of isokinetic strength could be used for the early detection of people at risk for developing NSLBP. However, these reference data correspond to subjects with CLBP, and were obtained from reviews in which no assessment of the quality of the evidence was carried out. This could limit our confidence in the reported data [31,32]. Furthermore, in CLBP, the evidence shows that pain and disability do have physical causes and have multifactorial etiology [33], with psychological factors [34], central sensitization [35] and kinesiophobia [36] playing a role in this type of patient. De Souza et al. [37] demonstrated that the peak torque of lumbar extensors in women with CLBP who have fear or negative beliefs related to the activity could be modified merely by using kinesiotape. This suggests that probably the strength values obtained in this type of patient may be influenced by other processes related to chronic pain and may not be an appropriate measurement on their own.

This allows us to question whether we estimate the ability to exert maximum trunk strength in subjects with chronic pain. Establishing whether an alteration in trunk muscle strength is present in those subjects who suffer from ALBP compared to healthy subjects is paramount in order to be able to develop training programs for preventing ALBP in the general population, and to manage this type of patient, avoiding its progression to CLBP. It is necessary to have data on the trunk’s isokinetic strength in patients with ALBP that will allow determination of which people are at risk for developing ALBP and thus prevent its appearance in healthy people. Moreover, this is necessary to manage it and avoid its progression to CLBP. Thus, the objective of this systematic review was (I) to determine the quality of evidence from studies assessing trunk isokinetic strength in subjects with ALBP compared to healthy subjects and (II) establish reference values of isokinetic trunk strength in subjects with ALBP.

## 2. Materials and Methods

The Preferred Reporting Items for Systematic Review and Meta-Analyses guidelines (PRISMA) were used [38] (Supplementary Table S1). The protocol of this review was registered in PROSPERO (CRD42020193458).

### 2.1. Study Search

Two authors (WR-F and DJ-M) conducted the search. The databases used were PubMed, Web of Science, Scopus and SPORTDiscus. The search was carried out from their inception to October 2020, the following keywords were included: “isokinetic”, “muscle strength”, “dynamometer”, “CORE”, “abdominal muscles”, “abdominal wall”, “torso”, “trunk”, “low back pain”, “low back ache” y “lumbago”. Search strategies are presented in Supplementary Table S2.

### 2.2. Eligibility Criteria

Articles that met the following criteria were included in this review. For aim (I): adult participants (age ≥18 years old), measures of isokinetic trunk flexors and extensors strength comparing a group of individuals with ALBP with a healthy control group, full-text available, and articles in English. For aim (II), the criteria for aim (I) were applied, but all the studies assessing isokinetic trunk flexors and extensors strength in individuals with ALBP, regardless of having a healthy control group or not, were included. Studies that only included either healthy people or subjects with chronic low-back pain were excluded.

### 2.3. Study Selection

Articles that were found eligible for inclusion in this review were entered into the Rayyan QCRI application, an app that assists in the article selection process, optimizing the screening time and allowing collaborative tasks (available for free at http://rayyan.qcri.org (accessed on 19 June 2020)) [39]. Duplicate references were removed, and two independent researchers (WR-F and DJ-M) reviewed titles and abstracts to identify articles met the eligibility criteria. The selected articles were then read in full, and the reference list was checked for relevant articles that could be included.

### 2.4. Assessment of the Risk of Bias and Quality of Evidence

Each article included in this systematic review was independently assessed for methodological quality and risk of bias by two researchers (WR-F and DJ-M). To the best of our knowledge, there is no scale for methodological evaluation adequate for the purpose of this review; therefore, we used the checklist proposed by Castro et al. [40], which combines some items from QUADAS [41] and a checklist to evaluate the methodological quality of both randomized and non-randomized studies of health care interventions [42]. This scale has 15 items divided into three sections (study sample, test procedures and data analysis, and results presentation). Each item was scored as “yes,” “no,” “unclear,” or “not applied”. A study was considered high risk of bias (low quality) when it received five or more “no” or “unclear” scores; in contrast, a study was considered low risk of bias (high quality) when it received less than five “no” or “unclear” scores. This cut-off score was determined on the basis of previous reviews that determined that 30% of negative results discriminate between studies of low or high methodological quality [43]. In case of disagreement among researchers, the consensus approach was used; for the case in which consensus could not be reached, a third researcher was consulted (LC-R).

### 2.5. Data Extraction and Analysis

The data extraction was performed by each researcher independently; the information extracted was related to the identification of the article (authors, year of publication, design and objective), the characteristics of the participants (total sample, gender, age, weight and height) and the isokinetic evaluation protocol (movement, position, range of movement, angular velocity, repetitions and contraction mode), in addition to results and main conclusions.

## 3. Results

### 3.1. Article Selection

**Figure 1:**
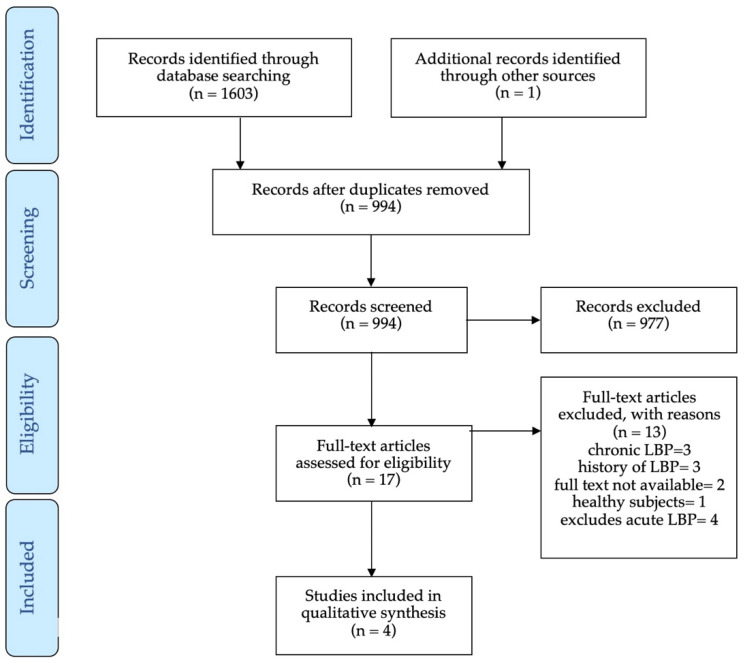
Flow chart for the systematic review.

No systematic reviews with a similar objective as the present study were found. From the initial search, a total of 1603 articles were retrieved (Figure 1), of which 610 were eliminated because they were duplicates. One additional article was identified from other sources. All the articles that assessed isokinetic trunk strength in individuals with ALBP presented a control group. Therefore, the number of articles included for aim (I) and aim (II) were the same. After evaluating titles and abstracts, 977 articles were excluded because they did not meet the inclusion criteria, leaving 17 articles for full-text analysis.

Of the 17 articles, two could not be retrieved because when contacting the authors, they did not have a digital copy to share due to the age of the publication (1982 and 1994). Of the remaining 15, after reading the full text, 11 articles were eliminated because they did not include the evaluation of subjects with ALBP. Thus, four articles were selected, and their reference lists were checked, and there were no new articles found.

### 3.2. Characteristics of the Studies

Table 1 presents the main characteristics of the included studies. One study [44] divided patients according to the duration of symptoms as acute and chronic, two [45,46] did so in acute, subacute and chronic, and only one [47] considered only subjects with acute pain. The number of participants with ALBP ranged from 21 to 46 subjects; Gabr et al. [46] do not indicate the exact number of ALBP subjects enrolled in their study. Age was not specified in the ALBP group in three of four studies, with only Hupli et al. [47] reporting an average age of 40.1 ± 8.9 years for men and 43.5 ± 9.2 for women. The physical activity profile was reported in only one study [47], but it does not specify which tool is measured.

Regarding the isokinetic dynamometer used, Suzuki et al. [44] used Cybex II, Akebi et al. [45] did not specify it, Hupli et al. [47] compared two dynamometers: Ariel 5000 (Ariel dynamics Inc., Trabuco Canyon, CA, USA) and Lido Multi-Joint II (loredan Biomedical, Inc., West Sacramento, CA, USA), while Gabr et al. [46] used biodex system 4 pro. Regarding the position in which the trunk strength was measured, three studies were performed in the standing position with knees in semi-flexion [45,46,47] and one study [44] used the supine position. In relation to the range of movement used, there was no concordance among the studies. One study used natural movement [47], another one 30° of flexion–extension [44], another one [45] a range of 0°–60° and another one the movement of maximum flexion and extension [46]. Three studies measured at velocities of 60°/s and 120°/s [45,46,47], while Suzuki et al. [44] used 30°/s.

The strength variables calculated were: (I) average peak torque (Nm), (II) trunk flexion (J), (III) trunk extension (J), (IV) abdominal strength (J), (V) average power, (VI) flexion–extension ratio (%) (flexion strength/extensor strength), (VII) fatigue (%) calculated as: (initial muscle strength–final muscle strength/initial muscle strength) x 100, and (VIII) Coefficient of Torque Variation (%).

### 3.3. Methodological Quality and Risk of Bias

In this review, 57 items (95%) were evaluated in the agreement between the two reviewers, the remaining three were decided by agreement (Table 2).

#### 3.3.1. Sample

Regarding the sample, three studies [44,46,47] describe the sample properly (item 1), however, only Gabr et al. [46] specified the inclusion criteria (item 2), none of the included articles explained how the sample size was calculated (item 3).

#### 3.3.2. Procedure

In relation to the trunk isokinetic evaluation procedure, two studies [45,47] report a familiarization process prior to measurement (item 4), none of them properly report the type or sequence of contraction only reporting the angular velocity used (item 5), only one study [47] reports a randomized order in the evaluations (item 6), and none of the four inform of the dominance of the extremities (item 7) which was evaluated as “not applied” because it is the trunk. All four studies [44,45,46,47] correctly describe the assessment position, the movements, and the form of stabilization used (item 8). None of the studies specify whether or not the same encouragement was given to each participant during the assessment (item 9). Considering the data analysis, only Hupli et al. [47] report that the Lido dynamometer software compensates for gravity, while Ariel does not (item 10); it is not clear if the other three studies performed gravity correction. Regarding the dependent variable, two studies clearly describe how the data extraction was performed [46,47], while, in the other two studies [44,45], it is not clear how data such as fatigue or the coefficient of variation were determined. None of the studies clarify whether the data were extracted from the isokinetic load range (item 12), and none report reliability measures, such as the intra-class correlation coefficient or standard error measurement (item 13).

#### 3.3.3. Presentation of Results

Regarding the presentation of results, three studies [44,46,47] adequately presented the results (item 14) and two [45,46] properly presented the inferential statistics (item 15). In summary, the four studies showed a high risk of bias [44,45,46,47].

### 3.4. Trunk Strength Parameters

Only two studies [46,47] measured peak torque in a similar way (Table 3).

#### 3.4.1. Average Peak Torque in Flexion and Extension

Two studies [46,47] determined the average peak torque in flexion and extension in healthy subjects and those with ALBP, measuring in standing, concentric mode, at velocities of 60°/s and 120°/s. Hupli et al. [47] compared men and women with ALBP and healthy subjects using two dynamometers, finding small, non-statistically significant differences between groups. Gabr et al, [46] when comparing men with ALBP and healthy controls, found significant differences in the peak torque of flexors (*p* = 0.004) and extensors (*p* = 0.003) at 60°/s and flexors (*p* < 0.001) and extensors (*p* < 0.001) at 120°/s, with an inverse F/E ratio at speeds of 120°/s in the ALBP group (Table 3).

#### 3.4.2. Coefficient of Variation

Akebi et al. [45] evaluated the relationship of the variability of the torque curves (CV) between subjects with ALBP and healthy controls finding CV values lower than the evaluation at 60°/s compared to 120°/s and, in addition, in both men and women the CV was lower in the control subjects compared to ALBP (Table 3).

#### 3.4.3. Average Power

Gabr et al. [46] found significant differences between average power in flexion (*p* = 0.004) and extension (*p* = 0.014) at 60°/s and between average power in flexion (*p* = 0.001) and extension (*p* = 0.045) at 120°/s between men with ALBP and a control group (Table 3).

### 3.5. Adverse Outcome from Trunk Isokinetic Assessment

From all the articles reviewed, none report adverse effects during trunk strength measurement using an isokinetic dynamometer in patients with ALBP. Suzuki et al. [44] and Gabr et al. [46] report that the assessment was performed without any complaints, Akebi et al. [45] do not report any undesirable effects during the assessment, and only Hupli et al. [47] reports a pain measured with visual analogue scale (VAS) (0–100) of 26.3 using the Ariel dynamometer and 15.2 with the Lido dynamometer in subjects with ALBP.

## 4. Discussion

The present systematic review was designed to (I) determine the quality of evidence from studies assessing trunk isokinetic strength in subjects with ALBP compared to healthy subjects and (II) establish reference values of isokinetic trunk strength in subjects with ALBP. The main findings of this study were (I) the articles included in this review present a high risk of bias; therefore, this indicates low quality of evidence, and (II) it was not possible to determine reference values, neither was it possible to determine whether trunk strength can distinguish between patients with ALBP and healthy subjects. However, based on data provided in the articles reviewed, we can report a range of peak flexion torque between 175. 1 Nm and 89.7 Nm at 60°/s and between 185 Nm and 81.5 Nm at 120°/s, and for peak torque in extension between 240.0 Nm and 91.5 Nm at 60°/s and between 217.5 Nm and 69.2 Nm at 120°/s in subjects with ALBP.

In addition to considering research with a low risk of bias, we should also consider studies with similar evaluation protocols to suggest reference values. Estrázulas et al. [48] after reviewing the literature, recommend reliable protocols for the evaluation of trunk flexors and extensors, carried out in a sitting position at velocities of 30°/s and 60°/s with a range of 30° (10° of flexion and 20° of extension) and/or in a standing position at velocities of 60°/s and 90°/s with a range between 90° and 95° of flexion and 15° of extension, both protocols in concentric mode with the axis in the anterior superior iliac spine. In the four studies reviewed, none included evaluation in sitting position; three of them [45,46,47] used the standing position but in a different range to the suggested and with velocities of 60°/s and 120°/s, which are commonly used in the measurement of trunk strength [48].

Concerning the variable analyzed, we know that the peak torque is widely used as a reference, allowing a direct comparison between studies. It has been previously used by Mueller et al. [31,49] to analyze subjects with low back pain, to determine deficits and to assess the effectiveness of training or therapy. In this review, two of the four included studies used peak torque in their analysis; however, Hupli et al. [47] found differences between the dynamometers used and therefore conclude and recommend that these data should not be compared among themselves. Analyzing this same variable, Gabr et al. [46] found that, unlike patients with CLBP, patients with ALBP have a significant reduction in the strength of trunk flexors and extensors, with an inverse F/E ratio at 120°/s, that is, greater than one, which indicates that the extensor muscles were mostly affected by the weakness. Mueller et al. [31] had previously reported the same, but with data from CLBP subjects, where a greater decrease in extensors’ strength was observed than in flexors, so the F/E ratio was higher than in healthy subjects. It is important to note that only the 60°/s flexor values obtained by Gabr et al. [46] are similar to those described by Mueller et al. [31] for the CLBP group, and those of 60°/s flexors in healthy subjects by Hupli et al. [47] with the control group by Mueller et al. [31]. On the other hand, Suzuki et al. [44] report differences in the strength of flexors and trunk extensors between ALBP and asymptomatic subjects, however, this variable was measured in Joules, which does not allow comparison, and also only indicates the existence of statistical differences, but does not report the *p*-value.

It is important to consider that none of the studies included in this review performed an isometric strength assessment of trunk flexors and extensors in subjects with ALBP using an isokinetic dynamometer. Among the studies reviewed, only Suzuki et al [44] evaluated isometric strength; however, they considered as a single group patients with acute and chronic pain (who had an average of eleven years of pain); so, unfortunately, it was not possible to distinguish isometric strength values in subjects with ALBP. Isometric evaluation is reliable in subjects with CLBP [50], and lower values of trunk strength have been observed in athletes and non-athletes with CLBP compared to healthy individuals. Cho et al. [16] propose that the risk for LBP and its severity would be associated with isokinetic weakness and the isometric weakness of trunk flexors and extensors.

From this, it is necessary to consider the importance of measuring these parameters in patients with ALBP, which could be used as an indicator of functionality or prognosis in these subjects, since isokinetic dynamometry has been widely used to evaluate the trunk strength but in patients with CLBP [50,51,52,53]; however, few studies have evaluated patients with ALBP.

Based on this systematic review, we cannot recommend reference values for the strength of trunk flexors and extensors in subjects with ALBP due to the high risk of bias of the articles included and the diversity of protocols, instruments and variables used in each article. Although three [44,45,46] of the four studies reviewed report differences in some strength parameters between individuals with ALBP and healthy subjects, these data are not confident given the limited quality of the evidence. Thus, it was also not possible to determine whether strength levels can help us distinguish between patients with ALBP and healthy subjects. On the other hand, we did not identify any studies that compared eccentric strength among these people. The eccentric contraction occurs when the external force is greater than the muscle strength, therefore, it plays an important role in the activities of daily life and sports, in the deceleration of the body during movements [54], so it would be interesting to investigate different types of contraction and eccentric/concentric ratio in subjects with ALBP compared to healthy subjects to understand the muscle dynamics of the trunk in different contexts or activities.

We can consider this review’s strength as having considered research with no prior date limit until 2020. However, that presents us with an associated difficulty since the studies we include have a range of 36 years of difference, time in which the standards of scientific publication have changed, and new guidelines have been developed [55,56], which could explain the high risk of bias found in this review.

In this context, it is necessary to conduct rigorous longitudinal studies, based on current methodological guidelines, that allow us to detect people at risk for developing ALBP and that consider the multiple aspects involved in LBP, both physical and psychological. For this reason, we can suggest the formation of working groups to determine consensus on the best way to approach the evaluation of this type of patient. For the reasons mentioned earlier, we consider it necessary to carry out new studies of high methodological quality that allow us to clarify if there are levels of strength associated with ALBP and to be able to prevent its appearance. In addition, given the questions regarding the evaluation of unnatural movements or those that do not necessarily represent the physiology or velocity of the movement performed on the isokinetic dynamometer [57], it is necessary to develop new technologies [28,58] that allow the evaluation of trunk strength related to a functional or athletic context that mimics the functional demands of the athlete or patient.

## 5. Conclusions

The findings of this systematic review indicate that the quality of studies assessing isokinetic trunk strength in subjects with ALBP compared to healthy controls was weak. Moreover, the available data did not allow presentation of reference values in patients with ALBP. Future research of high methodological quality is needed to establish reference values of trunk isokinetic strength in subjects with ALBP and to determine the ability of trunk strength to discriminate ALBP patients from healthy individuals.

## Figures and Tables

**Table 1:** Characteristics of individual studies.

Study	Objective	Participants	Age, Weight and Height (Mean ± SD)	Movement, Position and ROM	Velocity and Repetitions	Contraction Mode	Measured Outcomes
Suzuki et al. [44]	To measure the muscle strength and fatigability of the trunk flexors and extensors in normal pain-free subjects and in patients with LBP and to determine the role of the trunk muscles in LBP syndrome.	LBP group: 90 men. Acute pain: 38 Control group: 50 healthy men.	LBP group: 29.7 ± 5.4 yrs., 61.1 ± 8.5 kg, 167.8 ± 4.6 cm. Control group: 28.3 ± 4.3 yrs., 59.8 ± 7.5 kg,167.8 ± 5.2 cm.	Flexion 1: Supine with hands behind the neck, with hips and knees extended. Flexion 2: supine, hands behind the head, hips and knees bent. Extension: prone arms at the sides. ROM: 30° flexion and extension.	30°/s 1 rep of 90 s	Isometric: no distinction according to duration of symptoms. Isokinetic: concentric, according to duration of symptoms (ALBP and CLBP).	Torque isometric (J); Trunk flexion (Joule), Trunk extension (J), abdominal strength (J)
Akebi et al. [45]	To examine the difference in coefficient of variance (CV) of isokinetic trunk strength between healthy subjects and LBP patients.	LBP group: 143 (93 men and 50 women) Acute pain: 46, men 29 and women 17. Subacute pain: 38 Chronic pain: 59 Control group: 200 healthy subjects (112 men and 88 women)	LBP group: Men 51 ± 15.7 yrs., women: 50 ± 14.7 yrs. Control group: Men 49 ± 15.5 yrs., women 51 ± 15.3 yrs. Weight and height not described.	Standing with knees in semi-flexion. ROM: 0°–60° flexion and extension.	60°/s 3 rep 120°/s 5 rep	Isokinetic: concentric.	Coefficient of variance (%)
Hupli et al. [47]	To compare of trunk strength measurements between two different isokinetic devices used in clinical settings	LBP group: 21 (11 men and 10 women). Control group: 20 healthy subjects (10 men and 10 women)	LBP group: Men 40.1 ± 8.9 yrs., 79.5 ± 9.4 kg, 177.6 ± 4.9 cm. Women: 43.5 ± 9.2 yrs., 66.0 ± 13.3 kg, 164.9 ± 6.4 cm. Control group: Men: 39.7 ± 7.6 yrs., 78.9 ± 5.6 kg, 180.5 ± 6.8 cm. Women: 43.2 ± 7.2 yrs., 65.5 ± 6.8 kg, 168.6 ± 5.2 cm.	Standing with knees in semi-flexion. ROM: natural movement from vertical to flexion that each subject could perform.	60°/s and 120°/s 5 rep.	Isokinetic: concentric.	Average peak torque (Nm)
Gabr et al. [46]	To check and compare the muscle torque and power velocity of the trunk muscles in healthy men and male patients with low back pain to detect the relationship between low back pain and trunk muscles strength in the absence of structural neurological lesions.	LBP group: 50 men. Does not specify number per acute, subacute and chronic group. Control group: 50 healthy men.	LBP group: 22.9 ± 3.4 yrs., 77.7 ± 21.1 kg, 170.6 ± 6.4 cm. Control group: 23.4 ± 3.9 yrs., 76.1 ± 15.5 kg, 170.6 ± 7.9 cm.	Semi standing position. ROM: adjusted to each subject for maximum flexion and extension.	60°/s and 120°/s	Isokinetic: concentric.	Peak torque, flexors/extensor ratio, average power of trunk flexor and extensor.

SD: standard deviation; ROM: Range of motion; LBP: Low back pain; yrs.: years; rep: repetitions; s: seconds; ALBP: acute low back pain; CLBP: chronic low back pain.

**Table 2:** Methodological quality of the studies included.

Studies	Items															Total of N/UC	Total RoB
	1	2	3	4	5	6	7	8	9	10	11	12	13	14	15		
Suzuki et al. [44]	**Y**	N	N	N	UC	UC	NA	Y	UC	UC	UC	UC	N	Y	UC	11	High RoB
Akebi et al. [45]	N	N	N	Y	UC	UC	NA	Y	UC	UC	UC	UC	N	UC	Y	11	High RoB
Hupli et al. [47]	Y	N	N	Y	UC	Y	NA	Y	UC	Y	Y	UC	N	Y	UC	7	High RoB
Gabr et al. [46]	Y	Y	N	UC	UC	UC	NA	Y	UC	UC	Y	UC	N	Y	Y	8	High RoB

Items considered for rating: 1. Was the study population adequately described (i.e., sex, age, body mass, body height, kind of physical activity/lifestyle (sedentary, athlete, level of physical activity))?; 2. Was the description of selection criteria presented?; 3. Was there justification of appropriate sample size (through calculation or guidelines)?; 4. Were warm-ups and a familiarization protocol performed?; 5. Were type of muscle action (i.e., concentric and eccentric), sequence of action (i.e., concentric–concentric, concentric–eccentric, eccentric–eccentric), and velocity of movement described?; 6. Was the order of tests (velocities and trunk) randomized or counterbalanced?; 7. Was the lower limb dominance considered?; 8. Was the standardization of positions, movements and stabilization performed and properly described?; 9. Did participants receive the same encouragement during the test?; 10. Was gravity correction considered?; 11. Were the outcome measures clearly described?; 12. Were data extracted from the isokinetic load range?; 13. Were measures of reliability (e.g., Intraclass correlation coefficients (ICC), Standard error of the mean (SEM)) presented?; 14. Were results clearly described?; 15. Were appropriate inferential statistics presented?. N: no; Y: yes; UC: unclear; NA: not applied; RoB: risk of bias.

**Table 3:** Isokinetic trunk strength in acute low back pain patients (ALBP) and healthy adults for trunk extension and flexion.

Movement	Position	Acute LBP Group (Mean ± SD)	Control Group (Mean ± SD)	Unit	Study
Flexion	Supine	71.20 ± 22.85 (J)	86.69 ± 27.66 (J)	Trunk flexion (J)	Suzuki et al. [44]
		49.7 ± 21.7	42.0 ± 21.7	Fatigue (%)	Suzuki et al. [44]
	Standing	Ariel: 60°/s: 175.1 ± 61.4 Nm 120°/s: 155.7 ± 58.3 Nm Lido: 60°/s: 165.2 ± 47.7 Nm 120°/s: 185.0 ± 54.0 Nm	Ariel: 60°/s: 171.3 ± 45.2 Nm 120°/s: 165.2 ± 47.2 Nm Lido: 60°/s: 168.4 ± 48.8 Nm 120°/s: 187.0 ± 61.7 Nm	Average peak torque (Nm)	Hupli et al. [47]
		60°/s: Men: 89.7 ± 34.5 Nm 120°/s: Men: 81.5 ± 34.9 Nm	60°/s: Men: 118.7 ± 37.1 Nm 120°/s: Men: 121.1 ± 39.7 Nm	Average Peak torque	Gabr et al. [46]
		60°/s: Men: 38.9 ± 19.7 120°/s: Men: 32.0 ± 24.9	60°/s: Men 56.0 ± 25.2 120°/s: Men 57.7 ± 36.5	Average Power	Gabr et al. [46]
		60°/s: Men: 12.2 ± 5.4 Women: 12.2 ± 7.1. 120°/s: Men: 20.4 ± 9.2 Women: 29.7 ± 15.5	60°/s: Men: 8.9 ± 6.5 Women: 9.5 ± 4.9. 120°/s: Men: 17.3 ± 6.2 Women: 21.1 ± 8.0	Coefficient of variance (%)	Akebi et al. [45]
Extension	Supine	132.98 ± 29.91	156.72 ± 37.66	Trunk extension (J)	Suzuki et al. [44]
		19.3 ± 13.2	17.2 ± 10.8	Fatigue (%)	Suzuki et al. [44]
	Standing	Ariel: 60°/s: 178.9 ± 55.2 Nm 120°/s: 165.6 ± 52.6 Nm Lido: 60°/s: 240.0 ± 85.4 Nm 120°/s: 217.5 ± 89.5 Nm	Ariel: 60°/s: 189.3 ± 49.4 Nm 120°/s: 182.4 ± 52.6 Nm Lido: 60°/s: 264.0 ± 73.1 Nm 120°/s: 249.5 ± 68.3 Nm	Average peak torque (Nm)	Hupli et al. [47]
		60°/s: Men: 91.5 ± 57.1 Nm 120°/s: Men: 69.2 ± 49.6 Nm	60°/s: Men: 141.0 ± 64.5 Nm 120°/s: Men: 125.5 ± 68.1 Nm	Average Peak torque [46]	Gabr et al. [46]
		60°/s: Men: 41.8 ± 35.2 120°/s: Men: 37.6 ± 37.1	60°/s: Men: 68.4 ± 47.6 120°/s: Men: 61.7 ± 59.0	Average Power [46]	Gabr et al. [46]
		60°/s: Men: 11.4 ± 6.9 Women: 11.6 ± 5.9 120°/s: Men: 21.9 ± 9.0 Women: 24.5 ± 14.1.	60°/s: Men: 8.0 ± 5.8 Women: 9.2 ± 5.3. 120°/s: Men: 16.6 ± 6.6 Women: 22.2 ± 9.0.	Coefficient of variance (%)	Akebi et al. [45]
Flexion–Extension ratio (%)	Supine	55.9 ± 18.8	57.2 ± 16.0	% Trunk flexion/extension (J)	Suzuki et al. [44]
Abdominal Strength	Supine	69.73 ± 24.13	79.04 ± 29.22	Joule	Suzuki et al. [44]

SD: standard deviation; s: seconds.

## Supplementary Materials

The following are available online at https://www.mdpi.com/1660-4601/18/5/2576/s1, Table S1: PRISMA 2009 check list, Table S2: Search strategy for each database and number of articles found.
