# Supplementary material for: Isokinetic Trunk Strength in Acute Low Back Pain Patients Compared to Healthy Subjects: A Systematic Review
Source: Int J Environ Res Public Health. 2021 Mar 4;18(5):2576. doi: 10.3390/ijerph18052576 (PMC7967351; doi:10.3390/ijerph18052576)
Supplement: Supplementary file 1 [file ijerph-18-02576-s001.zip › supplementary table 2.docx]

|  | **Keyword** | **Pubmed** | **WoS** | **Scopus** | **SPORTDiscus** |
| --- | --- | --- | --- | --- | --- |
| S1 | “Isokinetic” | 7590 | 9905 | 12227 | 6616 |
| S2 | “Muscle Strength” | 34518 | 29098 | 66395 | 21332 |
| S3 | “Dynamometer” | 7328 | 10890 | 20861 | 4791 |
| S4 | S1 OR S2 OR S3 | 41590 | 43086 | 87354 | 27474 |
| S5 | “Core” | 329572 | 829408 | 1124684 | 11922 |
| S6 | “Abdominal muscles” | 12481 | 2144 | 12868 | 1418 |
| S7 | “Abdominal wall” | 21891 | 17835 | 41208 | 247 |
| S8 | “Torso” | 9206 | 8396 | 13481 | 3125 |
| S9 | “Trunk” | 54231 | 61911 | 104253 | 7031 |
| S10 | S5 OR S6 OR S7 OR S8 OR S9 | 419635 | 916748 | 1280632 | 20868 |
| S11 | “Low Back Pain” | 34280 | 45563 | 60873 | 6004 |
| S12 | “Low Back Ache” | 49 | 63 | 71 | 5 |
| S13 | “Lumbago” | 1373 | 675 | 2048 | 75 |
| S14 | S11 OR S12 OR S13 | 35250 | 46191 | 62037 | 6055 |
| S15 | S4 AND S10 AND S14 | 292 | 529 | 619 | 163 |
|  | Total | 1603 | | | |
|  | Duplicated | 608 | | | |
|  | Total without duplicated | 995 | | | |

**Supplementary table S2.**

**Search strategy for each database and number of articles found**

Time interval: from their inception to October 2020
